# Efficacy and safety of Tonifying Qi and activating blood Chinese herbal prescriptions for myocardial infarction: Study protocol for a multi-centered RCT

**DOI:** 10.1097/MD.0000000000031680

**Published:** 2022-11-25

**Authors:** Juju Shang, Shenglei Qiu, Yingjie Zhou, Sina Liu, Zi Wang, Xiaolei Lai, Hongxu Liu, Mingxue Zhou, Zhenmin Zhang, Pengyu Liu, Fangfang Zhang, Xianghui Meng

**Affiliations:** a Department of Cardiology, Beijing Hospital of Traditional Chinese Medicine affiliated to Capital Medical University, Beijing, China; b South of Guang’anmen Hospital, China Academy of Chinese Medical Sciences, Beijing, China; c Beijing University of Chinese Medicine, Beijing, China; d Beijing Insititute of Traditional Chinese Medicine, Beijing, China; e Capital Medical University, Beijing, China.

**Keywords:** acute myocardial infarction, heart failure, protocol, Tonifying Qi and activating blood circulation, traditional Chinese medicine

## Abstract

Acute myocardial infarction (AMI) is a common cause of death worldwide and heart failure (HF) is the main complication. Although the increase in percutaneous coronary intervention and drug treatment can reduce in-hospital mortality after AMI, the incidence of HF after AMI and the resulting risk of death are still rising, which causes difficulties in the rehabilitation of AMI patients after reperfusion.

**Methods:**

In this prospective, multicenter, randomized, double-blind, double-dummy, placebo-controlled trial, we will assigned 673 eligible patients with AMI after reperfusion into 4 groups: receiving Nao-Xin-Tong capsule (NXT), Bu-Yang-Huan-Wu (BYHW) granule (BYHW), Yang-Yin-Tong-Nao granule (YYTN), or placebo. The course of treatment will be 3 months. The primary outcome is HF incidence within 180 days. Nao-Xin-Tong capsule, BYHW granule, and Yang-Yin-Tong-Nao granule are different traditional Chinese medicines used for tonifying Qi and activating blood (TQAB).

**Results:**

Three months of TQAB combined with Western medicine may reduce the incidence of HF after reperfusion of AMI and improve patients’ quality of life.

**Discussion:**

This study will provide an important basis for the application of traditional Chinese medicine in patients with AMI after reperfusion and provide an evidence-based basis for the prevention and treatment strategy of HF after AMI.

## 1. Introductions

The morbidity and mortality rates associated with acute myocardial infarction (AMI) are still increasing. The data from the “Report on Cardiovascular Health and Diseases in China 2021” showed that the number of individuals with cardiovascular diseases has reached 330 million. The mortality rate of AMI is 121.59/100,000 among urban residents and that is 130.14/100,000 among rural residents.^[1]^ The China Patient-Centered Evaluative Assessment of Cardiac Events prospective cohort study showed that 2.5% of AMI patients experienced recurrence within 1 year after discharge, and the recurrence rate within 1 month after discharge was 35.7%.^[2]^ The readmission rate of AMI within 30 days was 6.3%, and nearly 50% occurred within 5 days after discharge. Of these patients, 77.7% were hospitalized because of cardiovascular events. The incidence of HF after AMI is 16.7%.^[3]^ The rehabilitation of patients with AMI after reperfusion is an urgent problem.

Traditional Chinese medicine (TCM) has a long history of being used to treat ischemic heart disease in China. The method of tonifying Qi and activating blood circulation (TQAB) is most commonly used. With the extensive development of AMI reperfusion, TQAB is often used as adjuvant therapy for AMI after reperfusion.^[4–6]^ Research has shown that TQAB can improve clinical symptoms and ventricular remodeling, reduce the occurrence of adverse cardiac events,^[7]^ and improve the quality of life of AMI patients after reperfusion.^[8]^ Our previous studies showed that TQAB can relieve chest pain and improve reperfusion injury in patients with ischemic heart disease. It can play a myocardial protective role by improving vascular endothelial function, alleviating abnormal vasoconstriction, inhibiting oxidative stress injury, and cardiomyocyte apoptosis.^[9–12]^ Moreover, TQAB can improve coagulation function in AMI patients.^[13]^

Many studies have shown that TCM has advantages in improving the clinical symptoms and disease prognosis.^[14]^ However, high-quality clinical evidence is lacking. In addition, the effects of TQAB with different drug components are consistent. However, there is a lack of relevant research on this topic.

This prospective, multicenter, randomized, controlled, double-blind research project aimed to assess the efficacy and safety of TQAB in patients with AMI after reperfusion. We’ll also evaluate whether the efficacies of the three commonly used drugs for TQAB are consistent.

This study was supported by the National Key Research and Development Project of China (grant number 2019YFC1708602), and was approved by the Ethics Committee of Beijing Hospital of TCM affiliated to Capital Medical University (No. 2020BL02-019-01).

## 2. Methods/design

### 2.1. Design

**Figure 1.**
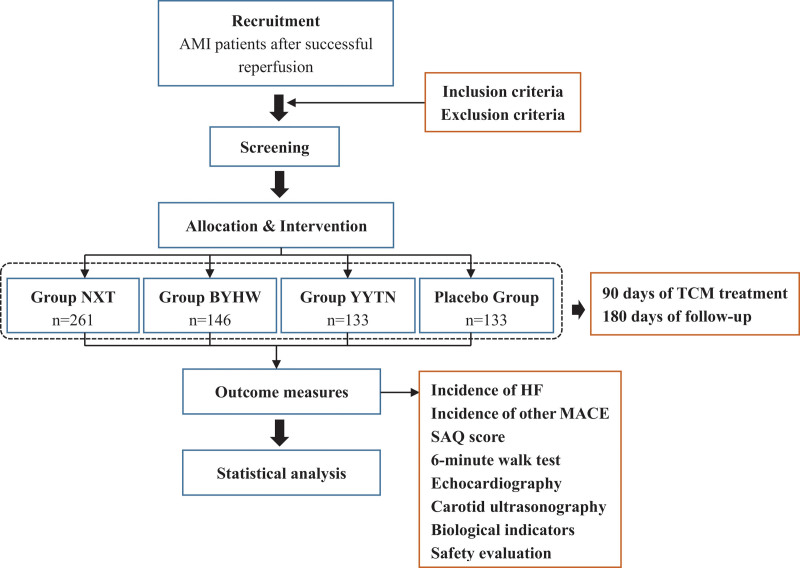
The flow chart of the study design. BYHW = Bu-Yang-Huan-Wu, HF = heart failure, MACE = major adverse cardiovascular events, NXT = Nao-Xin-Tong, SAQ = the Seattle angina questionnaire, TCM = traditional Chinese medicine, YYTN = Yang-Yin-Tong-Nao.

This is a prospective, multicenter, randomized, double-blind, double-dummy, placebo-controlled trial. It will be conducted at the Beijing Hospital of TCM affiliated to Capital Medical University and member units of the Beijing Office of Cardiovascular Disease Prevention and Treatment from December 2019 to December 2022. We will recruit 673 patients with AMI after reperfusion and assign them to Nao-Xin-Tong (NXT), Bu-Yang-Huan-Wu (BYHW), Yang-Yin-Tong-Nao (YYTN), or control groups. All participants will receive basic drug treatment according to the 2018 Guidelines for the Diagnosis and Treatment of AMI with Integrated Traditional Chinese and Western Medicine.^[15]^ The study drugs will be administered for 90 days. The patients will be followed-up for 180 days. The flowchart of the study design is shown in Figure 1.

### 2.2. Ethics approval

The trial was registered on June 18, 2021, in the Chinese Clinical Trial Registry (registration no. ChiCTR2100047435) and was reviewed and approved by the Ethics Committee of Beijing Hospital of TCM, affiliated to Capital Medical University (No. 2020BL02-019-01). This trial will be conducted in accordance with the Declaration of Helsinki, and the protocol will follow the recommendations of SPIRIT 2013^[16]^ (see Additional file 1). All the participants provided written informed consent.

### 2.3. Recruitment

We plan to recruit 673 participants from the outpatient or inpatient wards of the Beijing Hospital of TCM affiliated to Capital Medical University and member units of the Beijing Office of Cardiovascular Disease Prevention and Treatment. The researcher will take the initiative to introduce this trial, and the participants will voluntarily participate in the project. After screening by a clinician according to the inclusion and exclusion criteria, patients will be enrolled.

### 2.4. Inclusion criteria

Patients who fulfilled all of the following inclusion criteria will be included in this study: diagnostic criteria for AMI^[17]^ and atherosclerotic type^[18]^; diagnostic criteria for^[19]^ Qi deficiency and blood stasis syndrome; 2 weeks after successful reperfusion; 18 to 80 years of age, including men and women; and agreed to participate and sign an informed consent form.

### 2.5. Exclusion criteria

Patients with one of the following criteria will be excluded: severe liver or kidney insufficiency (alanine aminotransferase, ALT > 3 times the normal level; serum creatinine > 221 μmol/L); serious consumption state or malignant tumor; pregnancy or lactation; suspected or definite allergy to the test drug; participation in other clinical studies in the past 3 months; use of any preparation containing the same ingredients within 2 weeks; continuous mechanical adjuvant therapy after successful interventional therapy; and inability to actively cooperate with the treatment.

### 2.6. Withdraw criteria and management

Participants will be allowed or required to withdraw from the trial if they: develop a serious disease that is not suitable to continue in the investigator’s opinion; serious adverse events occur; and request to withdraw from the trial.

### 2.7. Randomization and grouping

A stratified random method will be adopted in the experiment with centers as the stratified factor. A total of 673 patients who fulfilled the criteria will be randomized into the NXT, BYHW, YYTN, and placebo groups in a 2:1:1:1 ratio. Randomized numbers will be generated by independent statisticians using SAS9.4 software (Beijing Hospital of TCM Version. Order number: 9C1XJD). Randomized numbers, rank sequences, and groupings will then be stored in opaque envelopes maintained by designers and statisticians throughout the study. All envelopes will be given running serial numbers, and each patient received one. Patients will be allocated to different groups according to the contents of the envelopes. Each participant received treatment assigned according to the randomization list. All clinical personnel and outcome assessors will be blinded to the intervention.

### 2.8. Blinding method

The double-blind and double-dummy methods will be used in this study. The first level of blindness will be the randomly coded group (groups A, B, C, or D) and the second level will be the treatment plan (experimental drug or placebo) for each group. The designer, researchers, and statisticians jointly will conduct the first unblinded and locked data analysis. Statistical analyses will be performed on the 4 sets of data under the supervision of an ethics committee. After the data analysis, a second uncovering will be conducted. The outcome information gatherers and outcome evaluators will be third-party statisticians who will not participate in study design or patient recruitment. The random coding table will be set up by the Clinical Trial Data Management and Statistics Unit, blind-sealed in duplicate, and stored by the designer and statistician in two locations for safekeeping. The entire drug-coding process will be documented by the compiler. That is, a blind record will be retained as one of the files of the clinical study. Blind emergency broken letters shall be kept by the clinical test unit and no one shall open them without permission. When serious adverse events occur during a study or when patients need to be rescued, it is necessary to know the patient’s treatment. Emergency blindness can be overcome by using trial centers. If patients experience severe adverse events, physicians should administer the relevant treatment. Emergency blindness will be determined by the principal investigator in the clinical trial unit. The reason, time, and location of blindness will be recorded in detail. The data remained intact even after blindness.

### 2.9. Intervention method

All patients will undergo basic treatment according to the 2018 Guidelines for the Diagnosis and Treatment of AMI with Integrated Traditional Chinese and Western Medicine. Drugs include antiplatelet agents (aspirin and clopidogrel), statins, beta blockers, nitrates, and angiotensin-converting enzyme inhibitors. Patients in the NXT group will be administered 4 Nao-Xin-Tong capsules and one bag of placebo granules thrice daily. Patients in the BYHW group will be administered one bag of BYHW granules and 4 placebo capsules 3 times daily. Patients in the YYTN group will be administered one bag of Yang-Yin-Tong-Nao granules and 4 placebo capsules 3 times daily. Patients in the placebo group will be administered one bag of placebo granules and 4 placebo capsules 3 times daily. The research drugs will be provided by Buchang Pharmaceuticals Limited Company. Placebo capsules and granules will be packaged in the same size, color, and taste as those of the study drugs. The treatment will last 90 days. The main components of the study drugs are listed in Table 1.

**Table 1:** The main components of the study drugs.

Drug	Chinese name	Latin name
Nao-Xin-Tong capsule	Shenghuangqi	Astragali Radix
	Chishao	Paeoniae Radix Rubra
	Danshen	Salviae Miltiorrhizae Radix et Rhizoma
	Danggui	Angelicae Sinensis Radix
	Chuanxiong	Chuanxiong Rhizoma
	Taoren	Persicae Semen
	Honghua	Carthami Flos
	Zhiruxiang	Olibanum
	Zhimoyao	Myrrha
	Jixueteng	Callerya reticulata (Benth.) Schot
	Niuxi	Achyranthis Bidentatae Radix
	Guizhi	Cinnamomum cassia Presl
	Sangzhi	Morus alba L
	Dilong	Pheretima
	Quanxie	Scorpio
	Shuizhi	Hirudo
Bu-Yang-Huan-Wu granule	Shenghuangqi	Astragali Radix
	Dangguiwei	Angelicae Sinensis Radix
	Chishao	Paeoniae Radix Rubra
	Dilong	Pheretima
	Chuanxiong	Chuanxiong Rhizoma
	Honghua	Carthami Flos
	Taoren	Persicae Semen
Yang-Yin-Tong-Nao granule	Shengdi	Rehmanniae Radix
	Shihu	Dendrobii Caulis
	Shenghuangqi	Astragali Radix
	Gegen	Puerariae Lobatae Radix
	Shuizhi	Hirudo
	Chuanxiong	Chuanxiong Rhizoma

### 2.10. Outcome evaluation

#### 2.10.1. Primary outcomes.

The primary outcome of this study is HF incidence. These data will be recorded over 180 days.

#### 2.10.2. Secondary outcomes.

1. Recurrence rates of other major adverse cardiovascular events, such as AMI, all-cause mortality, readmission rate due to cardiovascular events, non-fatal stroke, non-fatal AMI, and cardiovascular death, will be recorded by outpatient and telephone follow-up within 180 days.
2. The Seattle Angina Questionnaire score and 6-minute walk test results will be evaluated at baseline and on days 30, 90, and 180.
3. Echocardiography and carotid ultrasonography will be performed by the same physician at baseline and on days 90, and 180.
4. Biological indicators: cardiac troponin I, creatine kinase isoenzyme-MB, total cholesterol, triglyceride, low-density lipoprotein cholesterol, apolipoprotein A1, apolipoprotein B, D-dimer, prothrombin time, activated partial thromboplastin time, thrombin time, fibrinogen, international standardized ratio, platelets, and immunoglobulin G will be assessed at baseline and on days 90 and 180.

#### 2.10.3. Safety evaluation.

**Figure 2.**
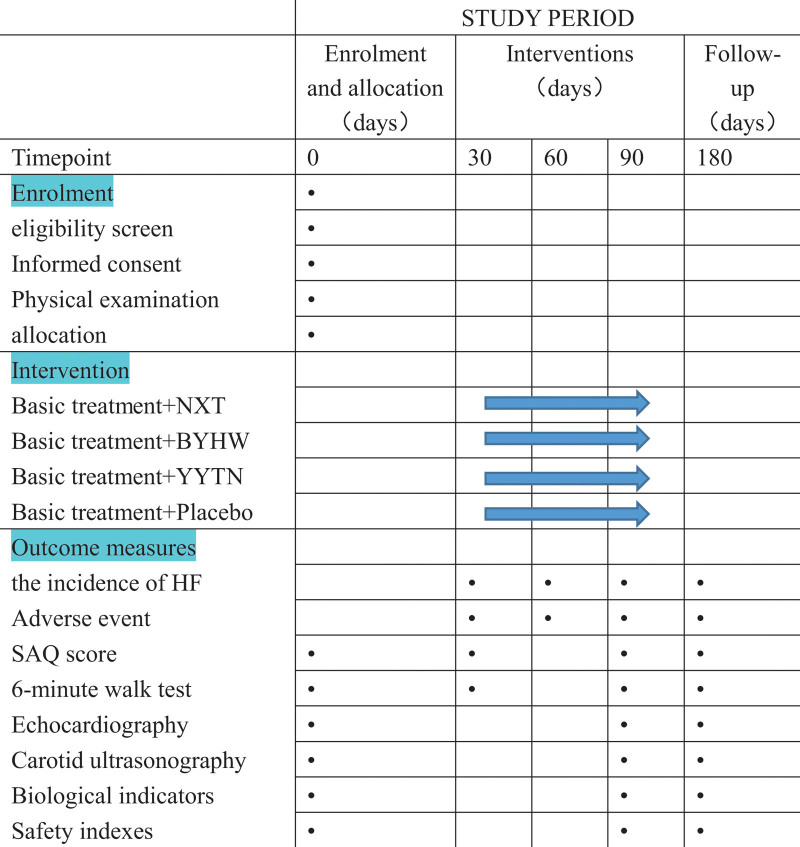
Spirit figure of enrollment, interventions, and assessments. NXT, patients will be given 4 Nao-Xin-Tong capsules and one bag of placebo granules 3 times daily; BYHW, patients will be given one bag of Bu-Yang-Huan-Wu granules and 4 placebo capsules 3 times daily; YYTN, patients will be given one bag of Yang-Yin-Tong-Nao granules and 4 placebo capsules 3 times daily. Placebo, patients will be given one bag of placebo granules and 4 placebo capsules 3 times daily. NXT = Nao-Xin-Tong, SAQ = the Seattle angina questionnaire, YYTN = Yang-Yin-Tong-Nao.

Safety will be assessed based on the time and severity of adverse events, vital signs, blood, urine, and feces routine, laboratory measurements (liver and renal function, electrolytes), and electrocardiogram. Adverse events were defined as any adverse events or any deterioration of existing diseases that occurred from the first administration of TQAB or placebo to 7 days after the last administration. The spirit figure of enrollment, interventions, and assessments are shown in Figure 2.

### 2.11. Biological samples preservation

Biological samples will be collected and preserved in each center.

### 2.12. Sample size

The sample size estimation was based on a superior efficiency comparison between the 2 groups of rates. The following formula was used for estimation:


$$\begin{array}{l} n_{T}+n_{C}=(\frac{n_{T}\pi_{T}+n_{C}\pi_{C}}{n_{T}+n_{C}})(1-\frac{n_{T}\pi_{T}+n_{C}\pi_{C}}{n_{T}+n_{C}}){\left[\frac{\mu_{1-\alpha }+\mu_{1-\beta }}{\pi_{T}-\pi_{C}}\right]}^{2} \end{array}$$

$n_{T}$ and $n_{C}$ are the sample sizes for the treatment and control, respectively; α is the test level; β is the type-II error; 1-β is the power; and μ is the standard normal deviation threshold (μ1-α and μ1-β represent the corresponding one-sided bounds of 1-α and 1-β, respectively).

Assuming that the sample size ratio between the treatment and control groups is *r*, the above sample size formula is reduced to


$$\begin{array}{l} n_{T}=(\frac{\pi_{T}+\pi_{C}/r\;}{1+1/r\;})(1-\frac{\pi_{T}+\pi_{C}/r\;}{1/r\;}){\left[\frac{\mu_{1-\alpha }+\mu_{1-\beta }}{\pi_{T}-\pi_{C}}\right]}^{2} \end{array}$$

If π_T_ and π_C_ are the endpoints of observation of the efficacy of secondary prevention of myocardial infarction in the NXT and control groups, the incidence of endpoints was 5.6% and 15.4%, respectively. The Bonferroni adjustment was used to adjust α, α = 0.05/6 (unilateral), and β = 0.2. The NXT group consisted of 261 patients.

If π_T_ and π_C_ are the total response rates of myocardial infarction in the BYHW and control groups, respectively, the total effective rates were 92.30% and 76.92%, respectively. The BYHW group consisted of 146 patients.

If π_T_ and π_C_ are the total response rates of myocardial infarction in the YYTN and control groups, respectively, the total effective rates were 93.88% and 78.43%, respectively. The YYTN group consisted of 133 patients.

According to the Drug Registration Administration Measures and considering factors such as falling off, 673 patients are required.

### 2.13. Statistical analysis

Relevant logic verification will be conducted to determine the causes of participants’ falling off and missing data.

Statistical analysis will be performed using SPSS 15.0 (PN:32119001, SN:5045602) by a third party that has no access to the blinding codes. Continuous data will be expressed as mean ± standard deviation. Categorical variables will be expressed as frequencies (%). Continuous variables between the groups will be compared using the *t* test for normally distributed values. A completely randomized design and one-way analysis of variance will be used to compare normally distributed continuous variables with homogeneity of variance between groups, a completely randomized design and one-way analysis of variance will be used. The SNK-q test will be used for pairwise comparisons between groups. The rank-sum test will be used to compare non-normally distributed continuous variables between groups. Comparisons between groups will be performed using the chi-square test (or Fisher’s exact test when the expected frequency is <5). Post hoc analysis was performed using LOGISTIC and COX regression analyses. All calculations will be performed using SPSS 15.0. The threshold for significance will be set to *P* < .05 (2-tailed).

### 2.14. Data management and quality control

All data will be obtained from the follow-up subjects. The data monitoring committee is composed of experts with relevant professional knowledge and experience. The data monitoring committee will regularly review the data accumulated in this study, track the progress of the project, collect the problems existing in the implementation of the project, and provide timely feedback to the project leader. The purpose is to protect the safety of the subjects and to ensure the reliability and effectiveness of the test. The investigators will maintain all trial data, including the confirmation of all participants, original informed consent forms, case report forms, and detailed records of drug distribution/recall. The subjects’ personal information will not be disclosed unless required by applicable law. Subject to confidentiality, the inspectors, ethics committee, research supervision, and management personnel have access to the original records of the subjects to verify the trial process and data. Except when permitted by the regulation, records of participants’ participation in the trials shall be kept confidential and shall not be made public. The data will be processed anonymously, omitting information that can identify the individual subjects. The trial data will be retained by the investigators. If the results of the trial will be published, the identity information of the participants would remain confidential.

## 3. Discussion

AMI is a serious threat to human health. Although reperfusion therapy is widely performed, heart failure, cardiogenic shock, arrhythmia, recurrent myocardial infarction, and rehospitalization after myocardial infarction still seriously affect the quality of life of patients and cause a huge economic burden to society. Therefore, it is important to reduce the occurrence of adverse events and improve patients’ quality of life.

TCM has a history of >2000 years of treatment for AMI. The TQAB method can improve cardiac function, slow myocardial remodeling, and improve the quality of life of patients.

We investigated the TCM treatment of 5000 AMI inpatients from 2000 to 2008 and found that TCM was helpful in improving the quality of life of AMI patients.^[20]^ Another clinical study has confirmed this conclusion.^[21]^

However, the effect of TQAB on the long-term prognosis of patients after myocardial infarction reperfusion requires high-quality evidence. Whether there are differences in the efficacy of different components of TQAB drugs requires further research.

Therefore, we adopted a prospective, multicenter, randomized controlled, double-blind, double-dummy method to observe the efficacy of TQAB with different components on the incidence of heart failure and quality of life after AMI reperfusion. To verify the scientific and universal applicability of TQAB in the treatment of AMI. Optimizing the treatment scheme of integrated traditional Chinese and western medicine for myocardial infarction after reperfusion. We are looking forward that the result of this study will provide a scientific basis for the clinical application of TQAB.

## Author contributions

**Conceptualization:** Juju Shang, Shenglei Qiu.

**Formal analysis:** Xiaolei Lai.

**Funding acquisition:** Juju Shang.

**Investigation:** Sina Liu, Zi Wang, Zhenmin Zhang, Pengyu Liu, Fangfang Zhang, Xianghui Meng.

**Methodology:** Juju Shang, Shenglei Qiu, Hongxu Liu, Mingxue Zhou.

**Project administration:** Juju Shang, Shenglei Qiu.

**Supervision:** Juju Shang, Shenglei Qiu.

**Writing – original draft:** Shenglei Qiu, Yingjie Zhou.

**Writing – review & editing:** Juju Shang, Shenglei Qiu.
